# Soft Ionic and Electronic Triboelectric Nanogenerators: Toward Attachable and Implantable Biomedical Applications

**DOI:** 10.1002/adma.202517321

**Published:** 2026-01-29

**Authors:** Kyongtae Choi, Jimin Park, Won Jun Song, Soohwan An, Seonggyu Choe, Byeong‐Rak Keum, Min Young Kim, Dae Hee Hwang, Young‐Geun Park, Sung‐Won Kim, Sangyul Baik, Gwang‐Bum Im, Younghoon Lee

**Affiliations:** ^1^ Department of Mechanical Engineering Kyung Hee University Yongin Republic of Korea; ^2^ Department of Mechanical Engineering Massachusetts Institute of Technology Cambridge Massachusetts USA; ^3^ Center for Nanomedicine and Department of Anesthesiology Harvard Medical School Brigham and Women's Hospital Boston Massachusetts USA; ^4^ Schepens Eye Research Institute of Mass Eye and Ear Boston Massachusetts USA; ^5^ Department of Ophthalmology Harvard Medical School Boston Massachusetts USA; ^6^ Department of Oral Medicine Infection, and Immunity Harvard School of Dental Medicine Boston Massachusetts USA; ^7^ Renal Division, Division of Engineering in Medicine Department of Medicine Brigham and Women's Hospital Harvard Medical School Boston Massachusetts USA; ^8^ Department of Biology Massachusetts Institute of Technology Koch Institute for Integrative Cancer Research Cambridge Massachusetts USA; ^9^ Department of Orthopaedic Surgery Stanford University School of Medicine Redwood City California USA; ^10^ Department of Mechanical Engineering Sungkyunkwan University Suwon Republic of Korea; ^11^ Department of Cardiac Surgery Boston Children's Hospital Boston Massachusetts USA; ^12^ Department of Surgery Harvard Medical School Boston Massachusetts USA

**Keywords:** biomedical engineering, iontronics, soft materials, triboelectric nanogenerators

## Abstract

The growing societal demand for convenient and personalized healthcare solutions has driven significant progress in human‐interactive technologies. Soft electronics, with their extrinsic deformability, have sparked innovations in the design of stretchable and highly adaptable biomedical devices that enhance wearability. Despite these advancements, portable power sources remain a key limitation, constrained by short operating times and the inconvenience of frequent recharging or battery replacement. To overcome this hurdle, triboelectric nanogenerators (TENGs), which convert mechanical energy into electricity, have emerged as promising sustainable power sources, offering high efficiency, lightweight design, and self‐sustaining operation. Recent developments in integrating TENGs with ionic materials have enabled their use on or beneath the skin, allowing the harvesting of biomechanical energy that would otherwise be wasted to power healthcare devices. This review provides a comprehensive overview of attachable and implantable TENGs, classified by electronic and ionic materials, and examines their material choices, device structures, and operational mechanisms. This review further explores various sustainable biomedical applications, assessing the performance of these devices in both sustainable power sources and self‐powered physiological signal sensing. Finally, key future research directions are outlined, including sweat tolerance, skin compliance, AI‐enabled TENG biointerfaces, acoustic transparency, minimally invasive implantation strategies, and regulatory considerations for clinical translation.

## Graphical abstract

This review provides an overview of triboelectric nanogenerator (TENG)–based biomedical applications by classifying studies into electronic and ionic systems across attachable and implantable platforms. It summarizes key material choices, device structures, and working mechanisms that characterize current TENG‐based research, and outlines six future research directions toward next‐generation self‐powered biomedical applications.

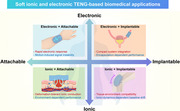

**Abbreviations**
A Am: acrylamide
ACMO: 4‐acryloylmorpholine
ChCl: choline chloride
DAAM: diacetone acrylamide
HDFS: heptadecafluoro‐1,1,2,2‐tetrahydrodecyl trichlorosilane
ITO: indium tin oxide
MPDMS: magnetic polydimethylsiloxane
PCL: polycaprolactone
PDMS: polydimethylsiloxane
PEG: poly(ethylene glycol)
PFA: perfluoroalkoxy alkane
PHBV: poly(3‐hydroxybutyrate‐co‐3‐hydroxyvalerate)
PMMA‐r‐PBA: poly(methyl methacrylate‐ran‐butyl acrylate)
PTFE: polytetrafluoroethylene
PVA: poly(vinyl alcohol)
TPU: thermoplastic polyurethane
VHB tape: very high bond acrylic tape
[DMIM]: 1‐dodecyl‐3‐methylimidazolium
[TFSI]: bis(trifluoromethanesulfonyl)imide.

## 1 Introduction

Growing societal interest in individual well‐being and the increasing demand for healthcare devices have led to rapid advancements in biomedical technology. Extensive studies in biomedical technologies have enabled more accurate measurement of health parameters, earlier disease detection, and highly personalized treatment than ever before. These biomedical devices have been broadly used for therapeutic applications such as physiological signal monitoring (e.g., heart rate, respiration, and blood pressure), wound healing, nerve stimulation, vascularization enhancement [1, 2, 3, 4]. However, integrating such biomedical devices into the human body still poses several challenges due to limited sustainable power supply for long‐term operation [5, 6] and the inherently low mechanical compliance of rigid materials, which may result in discomfort or detachment during human movement. As a result, flexible and stretchable electronics, capable of closely conforming to biological tissues, have become essential for wearable and implantable biomedical devices [7, 8, 9, 10, 11].

These soft and flexible electronics can alleviate discomfort during long‐term monitoring and conform to the skin surface, thereby enabling more accurate measurement of physiological signals [12]. Stretchable artificial skin, in particular, has become essential technology in human‐machine interfacing and health monitoring by converting external mechanical stimuli such as pressure, strain, and temperature into electrical signals [13, 14, 15]. Recent studies have focused on soft and stretchable devices incorporating ionic materials [16, 17, 18, 19, 20], in which ions serve as charge carriers. By replacing conventional electron‐based charge transport with ion transport, these ionic materials enable an intrinsically different mechanism of information processing based on ion migration, diffusion, and concentration gradients. Iontronics, which refers to a new concept of electronics, utilizes the migration of ions as charge carriers to enable functional signal and energy transfer. Recent advances in the field of iontronics and ionic materials have driven the development of soft devices that are highly stretchable, transparent, and biocompatible. Due to the ion transport mechanisms that are similar to those found in biological systems, ionic materials are suitable for biocompatible integration with human tissues.

While these advancements in soft electronics have significantly enhanced flexibility and mechanical compliance, conventional battery‐powered systems face multiple limitations [21, 22]. Specifically, conventional battery‐powered systems suffer from a short lifespan, frequent recharging requirements, discomfort due to rigid materials, and poor deformability for long‐term daily use. More importantly, device miniaturization increases reliance on external power sources for long‐term operation. To address these challenges, there have been significant advances in designing self‐powered energy harvesting systems that can harvest energy from the ambient environment or mechanical motion [23, 24, 25, 26, 27]. In recent years, triboelectric nanogenerator (TENG) based studies have demonstrated great potential as an innovative technology due to its high efficiency, lightweight, simple fabrication, and ability to convert mechanical energy into electricity [28, 29, 30, 31]. This energy harvesting technology aims to enable sustainable operation and eliminate the need for an external power source [32, 33], which is essential for the next‐generation of attachable and implantable biomedical electronics.

This review provides a comprehensive overview of TENG‐based attachable and implantable devices, with a particular emphasis on attachable and implantable biomedical applications that enable seamless human–machine integration (Table 1) [34, 35, 36, 37, 38]. We provide a focused analysis by categorizing TENG research into two distinct paradigms based on their charge carrier characteristics: electronic materials and ionic materials. A detailed examination is provided covering the material selection, device structure, and working mechanisms of TENG‐based devices. Furthermore, this review highlights various sustainable biomedical applications and the performance of devices in either self‐powered energy sources or physiological signal sensing. Finally, future research directions for next‐generation human–machine interfaces are discussed.

**TABLE 1:** Classification of biomedical devices and applications.

Working mechanism	Interface type	Functional module	Layer for contact electrification	Layer for electrostatic induction	Biomedical application	Refs.
Electronic	Attachable	Power source	PTFE (−)	Copper	Wound healing	[39]
			PCL (−)	Polypyrrole/polycaprolactone	Wound healing	[40]
		Self‐powered sensor	Chitosan‐diatom composite film (+)	Aluminum	Motion sensor	[41]
			PDMS (−)	Polyurethanes/silver nanowires	Pressure sensor	[42]
			PTFE (−)	Aluminum	Wearable sweat sensor	[43]
			PDMS (−)	Copper	Glucose biosensors	[8]
	Implantable	Power source	MPDMS (−)	Copper	Wireless power supply to implantable devices	[44]
			PFA (−)	Gold	Nerve stimulation	[45]
			PTFE (−)	Silver	Promoting bone regeneration	[46]
			PHBV/PEG:ChCl (+)	Magnesium	Promoting peripheral nerve regeneration and functional recovery	[47]
		Self‐powered sensor	TPU (+)	Silver nanowires	Real‐time ureteral peristalsis monitoring	[35]
			PTFE (−)	Gold	Battery‐free intracardiac pacemaker	[48]
Ionic	Attachable	Power source	VHB tape (−)	DAAM based ionogel	Voice‐monitoring sensor	[49]
			ITO‐coated PET film (−)	PMMA‐r‐PBA based ionogel	Skin‐attachable power source	[50]
			Ecoflex 00–30 (−)	AAm based hydrogel	Skin‐attachable power source	[51]
			HDFS coated silicone rubber (−)	AAm based organogel	Wound healing	[52]
		Self‐powered sensor	PDMS (−)	AAm‐based hydrogel	Flexible sensor for human motion caption	[53]
			Gelatin (−)	Agar‐based hydrogel	Motion sensing for infant care	[54]
			[DMIM]^+^[TFSI]^−^ ions based PDMS (+)		Grid‐free touch sensor	[55]
	Implantable	Power source	Silicone rubber (−)	AAm‐based hydrogel	Wearable and implantable power source	[56]
			Nylon (+)	PVA‐based hydrogel	Power source for implantable device	[57]
		Self‐powered sensor	Ecoflex 00–10 (−)	ACMO‐based organogel	Implantable ligament strain sensor	[58]

### 1.1 Mechanical Properties of Soft Materials

**FIGURE 1:**
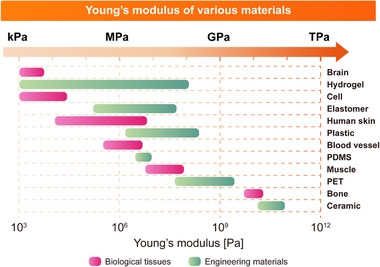
Range of Young's modulus values for biological tissues and engineering materials, from brain tissue at approximately 1 kPa to ceramics at 100 GPa [59, 67].

Soft materials are characterized by their intrinsically low elastic modulus and high mechanical compliance, enabling large deformation under subtle external stimuli (Figure 1). In contrast, conventional rigid materials possess high elastic moduli that are orders of magnitude higher than those of biological systems, including human skin, blood vessels, and muscles. This significant modulus mismatch at the tissue–device interface makes it difficult to achieve stable mounting and often triggers delamination during dynamic human motions. To overcome these limitations, soft materials have emerged as an essential alternative. Specifically, as their mechanical properties align with the low Young's modulus of biological tissues, soft materials‐based devices can achieve the seamless and stable integration required for diverse biomedical applications.

Among various soft materials, hydrogels have gained significant attention as key components for the fabrication of electronic and biomedical devices. Consisting of hydrophilic 3D polymer networks with 60%–90% water content [59], hydrogels exhibit desirable properties such as high transparency, stretchability, ionic conductivity, biocompatibility, and a tunable Young's modulus [60, 61, 62]. Their intrinsic softness and low elastic modulus, compared with conventional engineering materials, facilitate seamless integration with biological tissues. Owing to these characteristics, hydrogels provide a robust platform for designing highly deformable, skin‐compliant, and comfortable attachable and implantable devices, thereby facilitating seamless human–machine interfacing in biomedical applications [63, 64, 65, 66].

### 1.2 Working Mechanisms of Triboelectric Nanogenerator (TENG)

**FIGURE 2:**
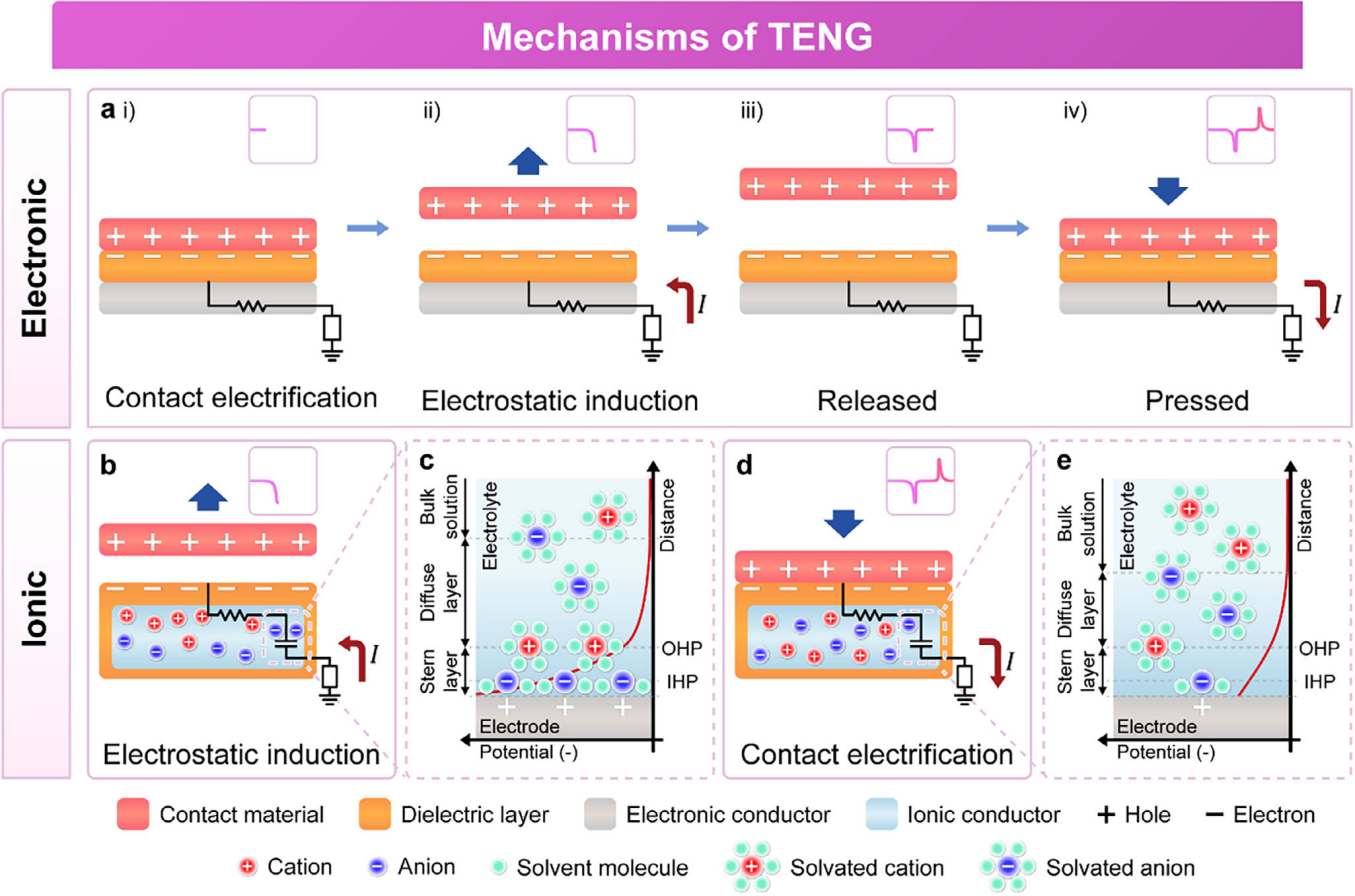
Working mechanisms of electronic (a) and ionic conductor‐based TENG (b‐e). (a) Electronic conductor−based TENG working mechanisms. (i) initial state of the electronic TENG. (ii) Contact electrification occurring between two contacting materials. (iii‐iv) electrostatic induction induced by surface charges during releasing and pressing. (b‐e) Ionic conductor−based fTENG working mechanisms. (b) Ion migration within the ionic conductor during release of the contact material, induced by electrostatic charges accumulated on the dielectric layer. (c) Schematic of the electrical double layer (EDL) at electrode‐electrolyte interface according to the Gouy−Chapman‐Stern model [84]. The inner Helmholtz plane (IHP) is defined by the centers of ions specifically adsorbed to the electrode surface, and the outer Helmholtz plane (OHP) is defined by the closest fully solvated ions [83]. (d) When the dielectric layer is pressed by the contact material, the cations induced by the surface charge are released. (e) This release subsequently triggers anion migration at the electrode–electrolyte interface, leading to a decay in the electrical potential.

As illustrated in Figure 2, the fundamental working mechanisms of TENG are based on two coupled effects called contact electrification and electrostatic induction [68, 69]. Contact electrification, which is triggered when the contact material and dielectric layer are pressed and released by external mechanical forces, results in the generation of positively and negatively charged surfaces as a result of differences in their electron affinity (Figure 2i) [70]. As two materials are separated, the charged surfaces generate a potential difference that induces the flow of electrons through the external circuit (Figure 2ii). When the contact materials and dielectric layer are fully separated, the system reaches electrostatic equilibrium (Figure 2iii). When the contact material is pressed, the induced electrons flow back from the ground to the electrode (Figure 2iv), generating alternating current under a periodic applied force.

In contrast to conventional electrodes, which are typically rigid, non‐transparent, and electron‐based, soft ionic materials possess unique properties such as intrinsic stretchability, transparency, and ionic conductivity arising from mobile ion transport [51, 53, 71, 72, 73, 74]. In an ionic material‐based TENG, electrostatic induction caused by periodic pressing of the contact material induces ion migration toward the electrode‐electrolyte interface within the ionic conductor (Figure 2b) [50, 75, 76, 77]. The ion migration driven by the negative charges accumulated on the dielectric layer attracts cations within the ionic conductor toward the surface of the dielectric layer and repels anions toward the electrode–electrolyte interface (Figure 2c) [78, 79]. The repelled anions induce electron flow through an external circuit, resulting in the formation of an electrical double layer (EDL) at the interface [80, 81], which behaves like a supercapacitor [82], as defined by the Gouy−Chapman−Stern model. The EDL consists of the Stern layer and the diffusion layer. The Stern layer comprises the inner Helmholtz plane (IHP) and the outer Helmholtz plane (OHP). The IHP is constituted by the centers of specifically adsorbed ions, whereas the OHP is constituted by the centers of the nearest solvated ions. When the dielectric layer is pressed by the contact material, the induced cations are released from its surface (Figure 2d). Consequently, anions are released at the electrode–electrolyte interface, causing a decay of the electrical potential (Figure 2e) [83]. Similar to the role of the electronic conductor in a conventional TENG, the ionic TENG paves the way for designing soft biomedical applications that facilitate safe, comfortable, and integrated human–machine interfaces.

### 1.3 Progress in Triboelectric Nanogenerators: From Portable to Implantable Devices

**FIGURE 3:**
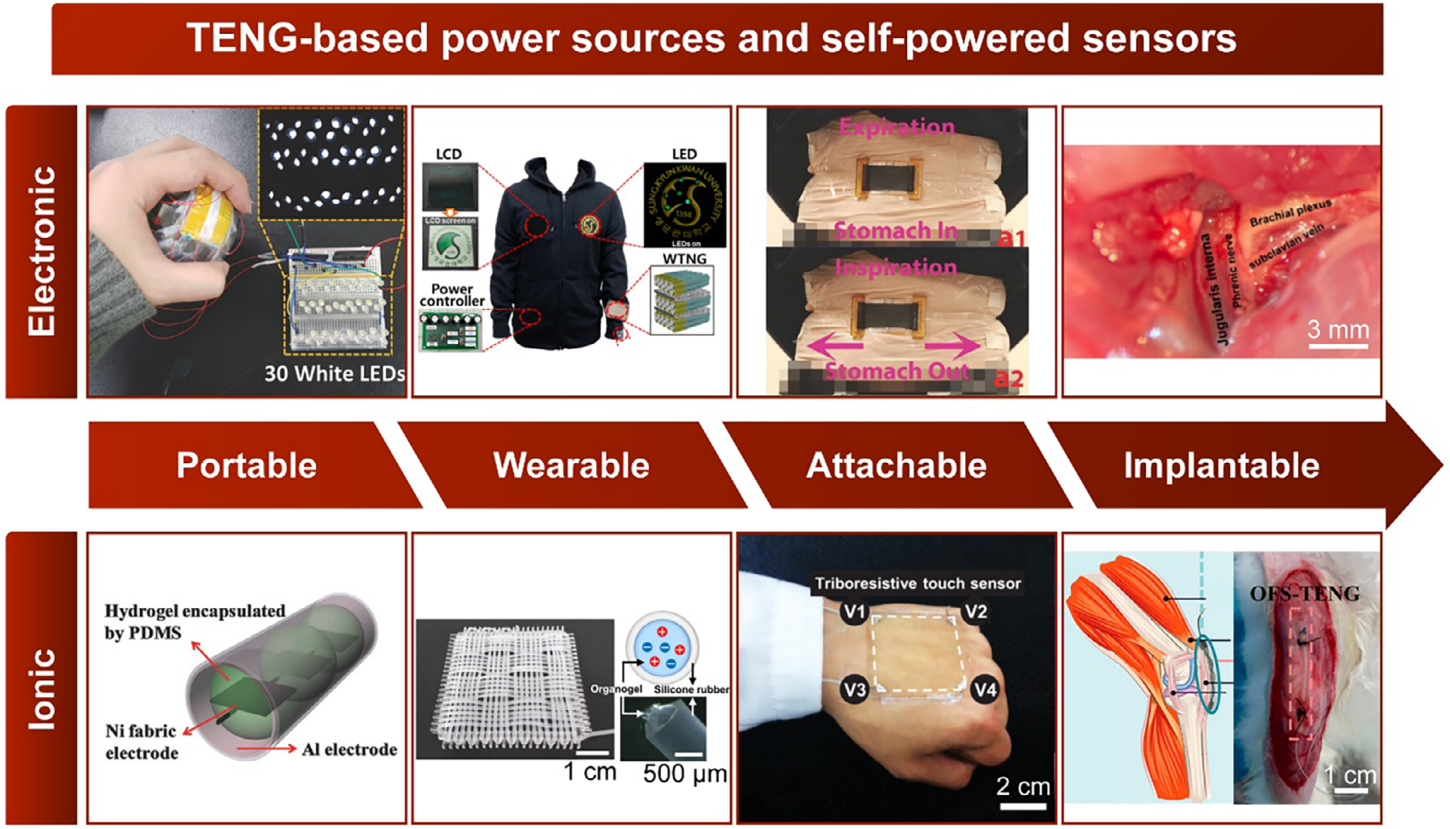
Representative electronic and ionic TENG devices designed for portable, wearable, attachable, and implantable applications. Reproduced with permission [85]. Copyright 2018, Wiley‐VCH. Reproduced with permission [86]. Copyright 2015, The American Chemical Society. Reproduced with permission [87]. Copyright 2015, Wiley‐VCH. Reproduced with permission [88]. Copyright 2024, The American Chemical Society. Reproduced with permission [89]. Copyright 2017, Wiley‐VCH. Reproduced with permission [52]. Copyright 2021, Wiley‐VCH. Reproduced with permission [55]. Copyright 2022, Wiley‐VCH. Reproduced with permission [58]. Copyright 2022, The American Chemical Society.

Since TENG was first reported in 2012, numerous studies have been conducted to integrate self‐powered capability into biomedical platforms, ranging from portable to implantable devices (Figure 3) [44]. TENGs offer distinct advantages, including a simple structure, efficient energy harvesting based on contact‐separation motion, and facile fabrication. In particular, the diverse material selectivity of TENGs enables the utilization of both electronic and ionic electrodes, leading to the development of transparent and intrinsically stretchable next‐generation healthcare devices. Initially, early studies focused on utilizing TENGs as power sources for portable and wearable applications by fabricating encapsulated architectures and wire‐type devices that could be woven into textiles. Moving forward, the skin‐attachable devices have advanced through extrinsic structural design (e.g., serpentine layouts, origami‐inspired patterns, island‐bridge arrays) and intrinsic material stretchability to conform to dynamic skin surfaces.

Recently, several innovative reports have indicated that the focus of TENG‐based biomedical devices is shifting toward implantable designs to overcome the limitations associated with external devices, particularly device‐induced user discomfort. This research trend aims to enable seamless, continuous, and personalized healthcare by reducing user discomfort through the implantation of devices within the human body. Tremendous advancements in device miniaturization, biocompatible materials, and precision fabrication methods have gradually enabled the development of integrated bio‐interfaces capable of stable interaction with the human body. Consequently, these next‐generation implantable TENGs are being actively developed to achieve reliable operation, sustainable energy harvesting, and real‐time physiological sensing within the human body.

## 2 Electronic TENGs for Attachable/Implantable Biomedical Applications

Electronic biomedical application systems are designed either to supply electrical energy for driving biological devices or to measure physiological signals and biomechanical movements. These applications can be broadly categorized into two types: electronic power sources that generate and deliver electrical energy, and electronic sensors that detect and quantify physiological signals [90]. Among them, TENG‐based power sources are lightweight and flexible, and have a capacity to harvest energy from subtle biomechanical movements such as motion, pressure, and vibration [91]. TENG‐based sensors convert the harvested energy into voltage signals, which can then be converted into quantitative data, enabling self‐powered sensing [92, 93, 94]. Owing to these advantages, TENG‐based power sources and sensors are considered promising candidates for biomedical applications [95]. They are classified into attachable and implantable systems based on their mode of integration. This section summarizes their respective operating environments, structural configurations, working mechanisms, experimental validations, performance (or sensing performance), and application scenarios. Finally, remaining challenges and future research directions will be discussed.

### 2.1 Electronic TENG‐Based Power Sources for Attachable/Implantable Biomedical Applications

Electronic TENG‐based power sources were initially developed to harvest biomechanical energy from daily human motion, offering a battery‐free power solution for wearable biomedical devices. Early efforts primarily focused on achieving stable electrical output and reliable energy harvesting performance. To improve wearability, structural stretchability strategies, such as serpentine interconnects and flexible substrates, were introduced [96]. Despite these advances, however, the intrinsic rigidity of electronic materials persists, causing mechanical mismatch and long‐term durability issues when interfaced with deformable skin. Nevertheless, leveraging their relatively high output power, recent studies have sought to extend electronic TENGs beyond passive energy harvesting toward active systems that directly translate the generated electrical output into therapeutic functions at the skin interface.

**FIGURE 4:**
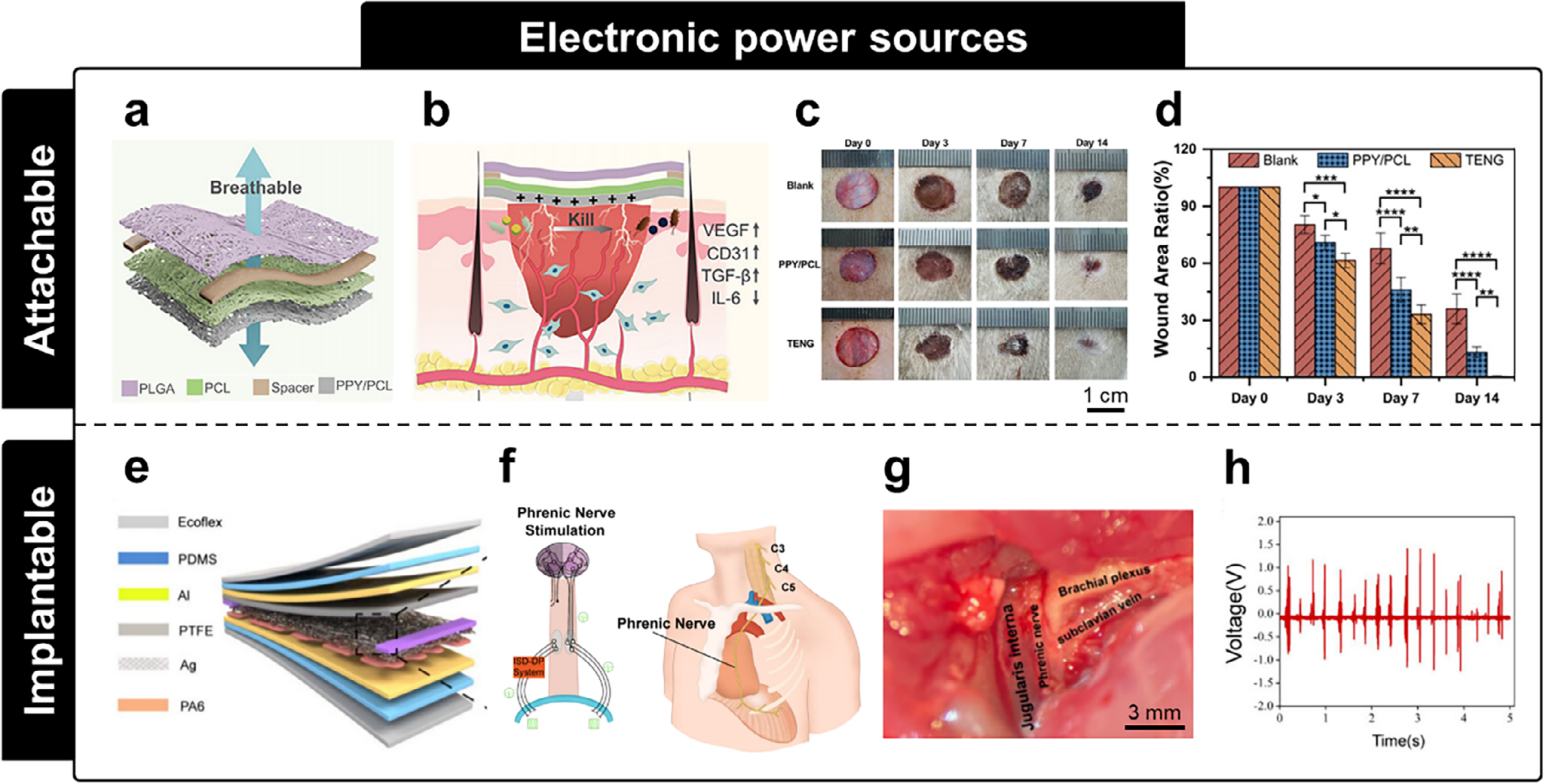
Electronic TENG‐based power sources for attachable/implantable biomedical applications. (a) Structural schematic of an attachable, self‐powered, and intrinsic bactericidal patch based on a TENG. Reproduced with permission [40]. Copyright 2023, The American Chemical Society. (b) Schematic illustration of synergistic bactericidal effect via membrane disruption induced by self‐generated electrical stimulation arising from mechanical motion and charge accumulation on polypyrrole (PPY). (c) Photographs of wound healing progression in the blank, polypyrrole/polycaprolactone (PPY/PCL), and TENG groups from day 0 to day 14 post‐treatment, and (d) wound area statistics at different periods. (e) Structural schematic of an implantable microvibration triboelectric nanogenerator (MV‐TENG). Reproduced with permission [88]. Copyright 2024, The American Chemical Society. (f) Schematic illustration of the mechanism of MV‐TENG‐driven phrenic nerve stimulation for diaphragm contraction and respiratory support in spinal cord injury (SCI) conditions. (g) Photograph of the surgical procedures. The cuff electrode was secured around the phrenic nerve. (h) In vivo output performance of the MV‐TENG subcutaneously implanted into the chest wall on the intact side of the rat, evaluated at 4 weeks post‐implantation.

This technological transition is exemplified by self‐powered systems designed for chronic wound healing and infection elimination, utilizing energy harvested from various biomechanical motions including joint movement, muscle contraction, and external touch [41, 42, 43, 97]. As shown in Figure 4a–d, this section introduces flexible, breathable, and self‐powered patch assembled from electrospun polymer triboelectric layers and polypyrrole (PPY) electrode for infected chronic wound healing [40]. This patch is a TENG‐based self‐powered system that promotes chronic wound healing and eliminates over 96% of bacteria through electrical stimulation and intrinsic antibacterial functionality, presenting an effective electrotherapy strategy [98, 99]. Figure 4a shows the structural configurations of the patch. The bactericidal patch is composed of polylactic‐co‐glycolic acid (PLGA), polycaprolactone (PCL), and PPY/PCL layers that are assembled sequentially from top to bottom. These elements function as current collectors, electron acceptors, and electron donors, respectively. The PCL and PLGA films are fabricated by electrospinning to form a triboelectric layer. The PPY/PCL is a layer of PPY deposited on top of another PCL film to form an electrode. The lower electrode, designated as the PPY/PCL layer, is the component that is affixed to the skin. Figure 4b shows the working mechanism of the patch. The patch is based on the vertical contact‐separation mode for the purpose of harvesting energy. The converted electrical stimulations have been shown to possess antibacterial properties while simultaneously reducing pro‐inflammatory cytokines such as IL−6, and increasing the expression of substances that promote wound healing, including angiogenic factors (VEGF), cell growth factors (TGF‐β), and marker of angiogenesis (CD31). The antibacterial mechanism of this patch involves two synergistic pathways. First, the positively charged PPY molecules electrostatically interact with the negatively charged bacterial cell membranes, leading to membrane disruption and bacterial death. Second, the electrical stimulation generated by the TENG produces a low‐intensity electric field that activates bacterial autolysis‐related genes, resulting in additional membrane damage and bactericidal effects. The wound healing mechanism is attributed to the electrical stimulation induced by the TENG patch [100], which activates signaling pathways such as MAPK/mitogen‐activated protein kinase. This activation enhances the expression of growth factors including VEGF and TGF‐β, thereby accelerating cell proliferation, migration, and tissue regeneration at the wound site. Figure 4c shows the experimental validation of the patch. It illustrates the actual application of the patch to the wounds of diabetic rats. The experiment was conducted using three groups: the blank group, the PPY/PCL group, and the TENG group, and the healing progress was monitored over a period of 14 days. Figure 4d shows the performance of the patch. When the patch was applied to the wounds of diabetic rats, the wound area ratio defined as the current wound area relative to the initial wound area was 68 ± 8% in the Blank group, 46 ± 7% in the PPY/PCL group, and 33 ± 5% in the TENG group on day 7. On day 14, the wound area ratio was 36 ± 8% in the blank group and 13 ± 3% in the PPY/PCL group, while the TENG group was entirely covered with newly formed pink epidermis. These results indicate that the application of the patch significantly promoted wound healing.

Electronic TENG‐based power sources for implantable biomedical applications harvest energy from various types of biomechanical motions when implanted inside the body [101], including heartbeats [102, 103], vascular pulsation, diaphragmatic motion during breathing, and gastrointestinal peristalsis, as well as from externally applied stimuli such as ultrasound (US) or magnetic fields [46, 47, 57, 104, 105, 106]. As shown in Figure 4e,f, this section introduces an implantable self‐driven diaphragm pacing system based on a microvibration triboelectric nanogenerator (MV‐TENG) for phrenic nerve stimulation [88]. This study presents a self‐powered, implantable diaphragm pacing system based on the MV‐TENG that efficiently harvests biomechanical energy to stimulate the phrenic nerve and induce diaphragmatic contraction, offering a promising strategy for the treatment of diaphragm paralysis following spinal cord injury. Figure 4e shows the structure of the MV‐TENG. The MV‐TENG is composed of polytetrafluoroethylene (PTFE), polyamide 6 (PA6), and silver nanowire layers that are assembled sequentially from bottom to top. PTFE serves as the negative triboelectric material, while PA6 acts as the positive counterpart; the two films are closely laminated to maximize the triboelectric effect. The silver nanowire solution is spray‐coated onto the surface of the PA6 layer to enhance surface charge density and improve electrical output performance. The negative electrode is constructed by dividing an aluminum foil into four equal sections and attaching them to the back surface of the PTFE layer. The entire device is encapsulated with polydimethylsiloxane (PDMS) and Ecoflex layers, which improve corrosion resistance and biocompatibility, while also ensuring mechanical flexibility and durability. Figure 4f shows the mechanism of the MV‐TENG. The working mechanism of the MV‐TENG is based on a vertical contact–separation mode [107], in which the device is implanted into the chest wall to harvest biomechanical energy generated by the rhythmic pressure of the intercostal muscles during respiration. The harvested energy is directed through a power conversion module within the implantable self‐driven diaphragm pacing (ISD‐DP) system, where it is rectified and filtered before being stored in a capacitor. The stored electrical energy is then delivered to the phrenic nerve via a cuff electrode, thereby inducing contraction of the diaphragm that has been paralyzed due to cervical spinal cord injury (SCI). This mechanism enables effective respiratory support for SCI patients with impaired spontaneous breathing. Figure 4g shows the experimental validation of the MV‐TENG. The image shows the actual implantation of the MV‐TENG into the chest wall of a rat. Output voltages were monitored for 4 weeks, and experiments were conducted to evaluate the diaphragm contraction and discharge success rates of the ISD‐DP system in the acute phase (immediately post‐injury), subacute phase (7 days post‐injury), and chronic phase (14 days post‐injury). Figure 4h shows the output performance of the MV‐TENG. The MV‐TENG exhibited stable output voltages over a 4‐week period (0.99 ± 0.65, 1.35 ± 0.60, 1.31 ± 0.55, and 1.39 ± 0.71 V), indicating its capability for long‐term energy harvesting. The ISD‐DP system, combined with the MV‐TENG, demonstrated diaphragm contraction and discharge success rates of 70.27% in the acute phase, 98.39% in the subacute phase, and 90.44% in the chronic phase. These findings support the potential of the ISD‐DP system as a viable diaphragm pacing device.

TENG‐based electronic power sources have expanded their application beyond initial biomechanical energy harvesting. They are now successfully utilized in diverse advanced applications, including flexible patches for self‐powered electrotherapy, promoting chronic wound healing and sterilization, and implantable systems that have been successfully applied to diaphragm pacing to assist respiration in patients with diaphragm paralysis. This evolution significantly broadens their potential in both wearable and implantable biomedical fields.

### 2.2 Electronic TENG‐Based Sensors for Attachable/Implantable Biomedical Applications

The functionality of electronic TENG‐based systems has now transcended simple energy supply to enter the phase of active sensing. These sensors convert subtle biomechanical pulsations and vibrations into precise electrical signals, facilitating advanced diagnostic capabilities like cardiovascular monitoring and bladder volume detection. This signifies a crucial technological shift, moving from mere external observation toward the quantitative assessment of internal physiological dynamics. However, the inherent stiffness of the electronic components persists as a fundamental constraint, causing mechanical mismatch when interfaced with soft biological tissues.

**FIGURE 5:**
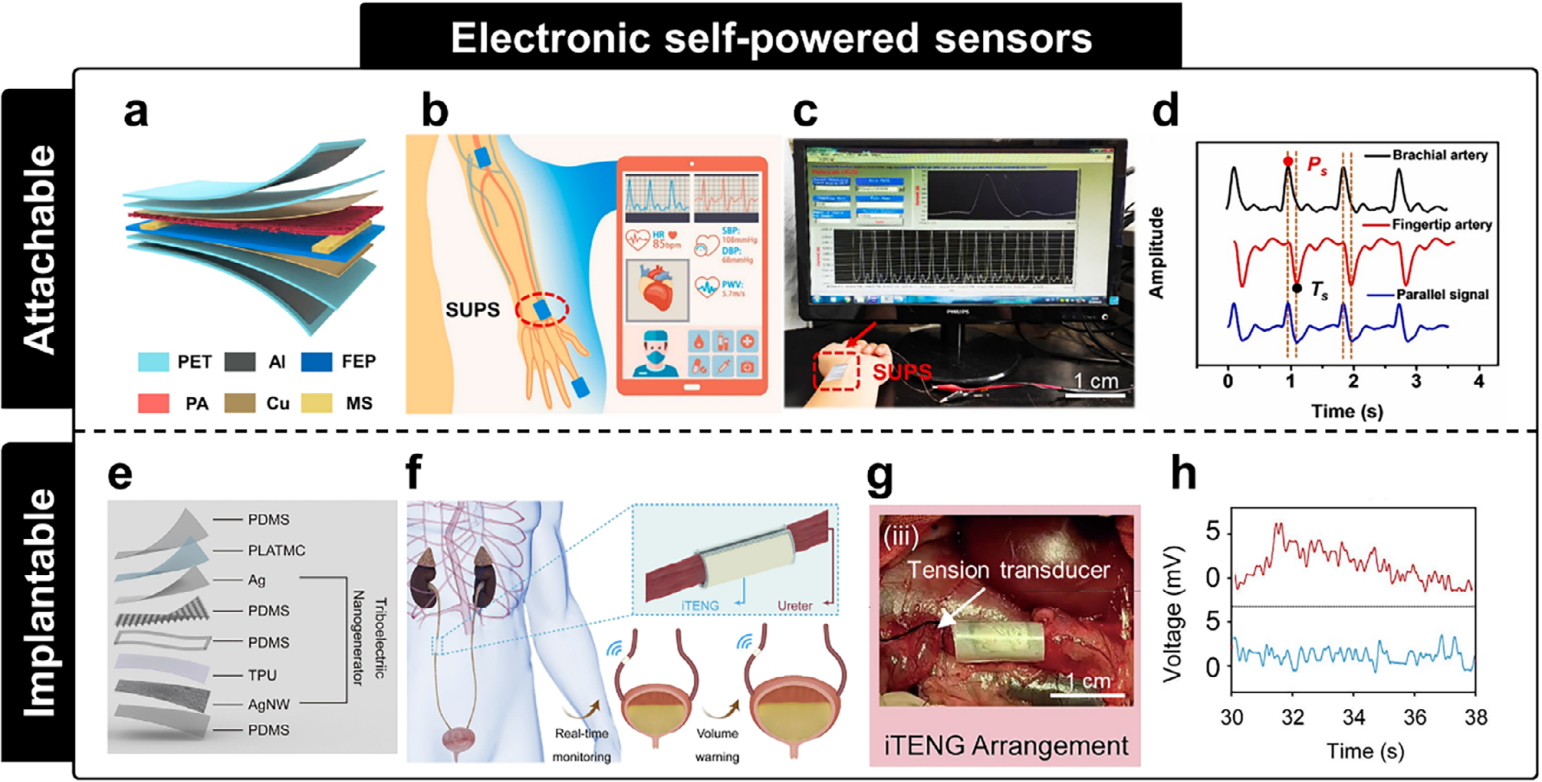
Electronic TENG‐based self‐powered sensors for attachable/implantable biomedical applications. (a) Schematic structure diagram of the self‐powered ultrasensitive pulse sensor (SUPS). Reproduced with permission [111]. Copyright 2021, Elsevier. (b) Illustration of the working mechanism of the SUPS for noninvasive multi‐indicators cardiovascular monitoring. (c) The real‐time pulse wave signal detected by the SUPS attached on radial artery. (d) A graph comparing the characteristic points such as the systolic peak (P_s_) and the second peak (T_s_) in the superimposed pulse wave signals acquired by attaching two SUPSs to the brachial and fingertip arteries with the corresponding points in the individual pulse wave signal. (e) Schematic illustration of the structure of implantable triboelectric nanogenerator (Imp‐TENG) based on shape memory polymer (SMP). Reproduced with permission [35]. Copyright 2024, Elsevier. (f) Illustration of the working mechanism of the Imp‐TENG for bladder volume monitoring. (g) Implantation of the tension transducer and the Imp‐TENG on the porcine ureter for bladder volume monitoring. (h) Monitoring of the peristaltic state and stationary state of the ureter using the Imp‐TENG.

These self‐powered devices, when attached to the skin, harvest energy from various types of subtle physiological signals and biomechanical movements such as pulse, respiration, voice‐induced vibration, muscle motion, and joint motion, and convert the resulting generated voltage into quantitative data [108, 109, 110]. As shown in Figure 5a–d, this section introduces a self‐powered ultrasensitive pulse sensor for noninvasive multi‐indicators cardiovascular monitoring [111]. A self‐powered ultrasensitive pulse sensor based on a triboelectric nanogenerator is developed for detection of pulse waves from various arteries, enabling noninvasive multi‐indicator cardiovascular monitoring. Figure 5a shows the structural configurations of the self‐powered ultrasensitive pulse sensor (SUPS). The SUPS has a multilayered structure composed of triboelectric layers, electrodes, a spacer, electrostatic shielding layers, an insulating layer, and an encapsulating layer. A nanowire‐structured fluorinated ethylene propylene film and a fibrous polyamide film are used as triboelectric layers, with copper foil attached to the backside of each layer as the electrode. A porous melamine sponge is placed between the two triboelectric layers as a spacer to enable contact–separation. Aluminum foil is attached to both sides of the SUPS as the electrostatic shielding layer, and polyethylene terephthalate (PET) film is inserted between the copper electrode and the aluminum foil as the insulating layer. The entire structure is encapsulated with PET film. Figure 5b shows the operating mechanism of the SUPS. The SUPS, when attached to the skin, harvests energy from arterial pulse movements and utilizes the resulting voltage signals for pulse monitoring. The SUPS calculates heart rate (HR) based on the interval between systolic peak (P_s_) within the pulse waveform, and enables noninvasive multi‐indicator cardiovascular monitoring by integrating multiple SUPSs distributed across different arterial pulse sites. Figure 5c shows the experimental validation of the SUPS. It demonstrates real‐time signal acquisition and monitoring with the SUPS attached to the wrist pulse site. The measured HR shows strong agreement with the results from commercial electrocardiograph (ECG) devices. Owing to its high measurement accuracy, the system was further tested for multimodal monitoring by simultaneously measuring pulse waves at both the brachial and fingertip arteries. Figure 5d shows the sensing performance of the SUPS. Measurements were conducted by connecting the SUPS to the brachial and fingertip arteries in a reverse parallel configuration. This connection inverts one of the pulse wave signals, allowing the ascending signal from the brachial artery and the descending signal from the fingertip artery to be superimposed. The resulting composite signal shows excellent agreement with the characteristic points of the individual pulse waveforms. Based on the measured time interval between P_s_, second peak (T_s_), and pulse transit time (PTT) are determined, which are then used to calculate the pulse wave velocity (PWV). Furthermore, the PTT value is applied to a correlation model with blood pressure (BP) to estimate both systolic and diastolic BP. The localized PWV values fall within the measurement range of clinical devices, and the estimated BP values satisfy the error tolerance defined by the standard of the Association for the Advancement of Medical Instrumentation. Together with accurate estimation of PWV and BP based on PTT, the reliability of heart rate monitoring further supports the potential of this system for human applications, suggesting its utility in the prevention and adjunctive treatment of cardiovascular diseases such as arrhythmia, arterial stiffness, and hypertension.

Electronic TENG‐based sensors for implantable biomedical applications harvest energy from various types of biomechanical motions when implanted inside the body, including heartbeats, vascular pulsation, diaphragmatic motion during breathing, and gastrointestinal peristalsis, and convert the resulting generated voltage into quantitative data [48, 112, 113, 114, 115, 116]. As shown in Figure 5e–h, this section introduces an implantable sensor based on shape memory polymers (SMP) and TENG: Monitoring ureteral peristalsis to assess bladder volume [35]. This sensor utilizes a body temperature‐responsive SMP as a support and enables early warning of bladder volume by monitoring ureteral peristalsis through implantable triboelectric nanogenerator (Imp‐TENG) implanted in pigs, suggesting its potential for clinical urological monitoring. Figure 5e shows the structure of the Imp‐TENG. The Imp‐TENG features a multilayered architecture comprising triboelectric layers, electrode layers, a spacing layer, a shape‐memory supporting layer, a shielding layer, and an encapsulating layer. The triboelectric layers are thermoplastic polyurethane film, each backed with either a silver electrode or silver nanowires. A PDMS‐based intermediate spacer is interposed between the two triboelectric layers to maintain an appropriate separation distance and facilitate stable contact–separation interactions. The SMP, poly(lactide‐co‐trimethylene carbonate), serves as an internal structural and restorative element, enabling the device to maintain its geometry and suppress mechanical interference under in vivo conditions. The entire device is encapsulated in the PDMS outer layer, which prevents infiltration from the surrounding liquid environment. Figure 5f shows the operating mechanism of the Imp‐TENG. The operating mechanism of the Imp‐TENG sensor is based on the vertical contact‐separation mode. The Imp‐TENG enables indirect evaluation of bladder volume by converting subtle pressure fluctuations caused by ureteral peristalsis into electrical signals. The acquired signals allow for quantitative analysis of peristaltic frequency and rate, and when combined with the estimated urine volume transported per contraction, facilitate high‐precision monitoring of bladder filling status. Figure 5g shows the experimental validation of the Imp‐TENG. For comparison, both a tension transducer and the Imp‐TENG were implanted on the ureter of a live pig. In the in vivo experiment, voltage signals generated by the Imp‐TENG were used to distinguish between peristaltic and stationary states of the ureter, and the results were compared with those from the tension transducer to verify the timing and accuracy of detection. Figure 5h shows the sensing performance of the Imp‐TENG. The blue graph represents the stationary state of the ureter when urine is not flowing into the bladder, during which the Imp‐TENG generates a low voltage. The red graph represents the peristaltic state when urine is actively transported through the ureter, during which the Imp‐TENG generates a higher voltage. The tension transducer also produced similar results at corresponding time points. These results indicate that the Imp‐TENG may serve as a low‐invasive and self‐powered option for bladder volume sensing.

### 2.3 Current Challenges in Electronic TENGs for Attachable/Implantable Biomedical Applications

Despite notable progress in this field, challenges related to electrode‐tissue interfaces and wiring remain major limitations, hindering seamless device integration and long‐term operational stability. Conventional electronic electrodes typically utilize rigid metallic materials (e.g., gold, platinum, or copper) due to their superior electrical conductivity. However, these materials exhibit a significant Young's modulus mismatch with soft biological tissues, often leading to stress concentration, chronic inflammation, and mechanical delamination at the interface. While alternative materials like carbon nanotubes, graphene, or silver nano wire offer improved flexibility, they still suffer from limited intrinsic stretchability or potential long‐term biocompatibility issues.

These material limitations are further compounded by wiring‐related issues. In attachable systems, external wiring is often required when the optimal site for energy harvesting—typically mobile regions such as the chest or joints—does not coincide with the therapeutic or sensing target, which may be located in static areas like the limbs. Similarly, in implantable systems, wiring is necessary for both power transfer and signal transmission. While stable energy must be harvested from large, involuntary motions like the heartbeat, the functional target (e.g., the bladder) is often located in regions with limited motion. This mismatch necessitates internal wiring to connect the TENG to dynamic sites, but conventional rigid metallic wiring often causes chronic immune responses, which make long‐term in vivo experimentation challenging. As a result, most current implantable TENG studies have been limited to short‐term validation (typically several weeks).

Overcoming the electrode and wiring issues arising from the tissue–device mismatch and inflammatory burden of rigid metallic components necessitates materials with intrinsic mechanical compliance and biocompatibility. As a potential solution to these issues, we propose the use of ionic conductors. Their inherent soft and stretchable characteristics offer a promising strategy to resolve these problems, highlighting their potential for realizing stable, long‐term implantation.

### 2.4 Summary of Electronic TENGs for Attachable/Implantable Biomedical Applications

This section summarizes electronic TENG‐based systems designed for attachable and implantable biomedical applications across two principal roles: sustainable power sources and self‐powered sensors that transduce various physiological activities. Representative demonstrations span from skin‐mounted patches and textiles for everyday motions to implantable devices driven by respiratory or cardiac dynamics for therapeutic support. The previous section addressed the inherent limitations of conventional electronics in biomedical applications. Building upon these considerations, the transition from rigid metallic electrodes to soft, biocompatible conductors is recognized as a key technical strategy to effectively bridge the gap between high electronic performance and long‐term biological safety. However, both the electronic TENG platforms and the ionic TENG platforms utilizing these soft conductors possess unique advantages alongside their inherent limitations. Therefore, before delving into ionic TENG‐based systems, Table 2 provides a concise comparison of electronic and ionic TENGs, detailing their respective strengths and limitations pertinent to biomedical applications.

**TABLE 2:** Comparison of key performance parameters of electronic and ionic TENG.

Key Attribute		Electronic TENG	Ionic TENG
Stretchability	Extrinsic (Structural)	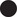	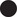
	Intrinsic (Material)	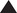	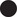
Transparency	Optical	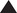	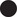
	Acoustic	X	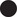
Compatibility	Environmental (Temperature/Humidity)	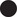	X
	Biological (Biocompatibility)	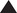	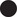
Conductivity	—	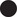	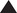
Affordability	—	X	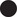

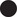
: Excellent / High |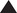: Moderate | X: Poor / Low / None

## 3 Ionic TENGs for Attachable/Implantable Biomedical Applications

Building upon the electronic systems discussed in the previous section, this section introduces ionic TENG‐based platforms designed for attachable and implantable biomedical applications [86]. These applications can be broadly categorized into two types: ionic power sources that generate and deliver electrical energy, and ionic sensors that detect and quantify physiological signals [116, 117, 118]. Due to their gel‐based composition, these systems offer high imaging invisibility and excellent biocompatibility, while maintaining the lightweight flexibility of their electronic counterparts [119]. Owing to these distinct advantages, this section summarizes their specific structural configurations and performance in dynamic physiological environments [120]. Finally, remaining challenges and future research directions will be discussed.

### 3.1 Ionic TENG‐Based Power Sources for Attachable/Implantable Biomedical Applications

While electronic TENGs remain a benchmark for high power output and structural stability due to superior electron mobility, ionic TENGs have emerged as a vital complementary advancement to bridge the mechanical gap between rigid devices and soft biological tissues [117, 118]. By utilizing hydrogels and organogels with tissue‐like Young's moduli, these ionic systems minimize inflammation and physical discomfort [79, 119, 120]. Furthermore, their inherent optical and acoustic transparency ensures compatibility with medical imaging (MRI/ultrasound), marking a significant milestone in achieving seamless biological assimilation without sacrificing the functional strengths of traditional electronic systems.

**FIGURE 6:**
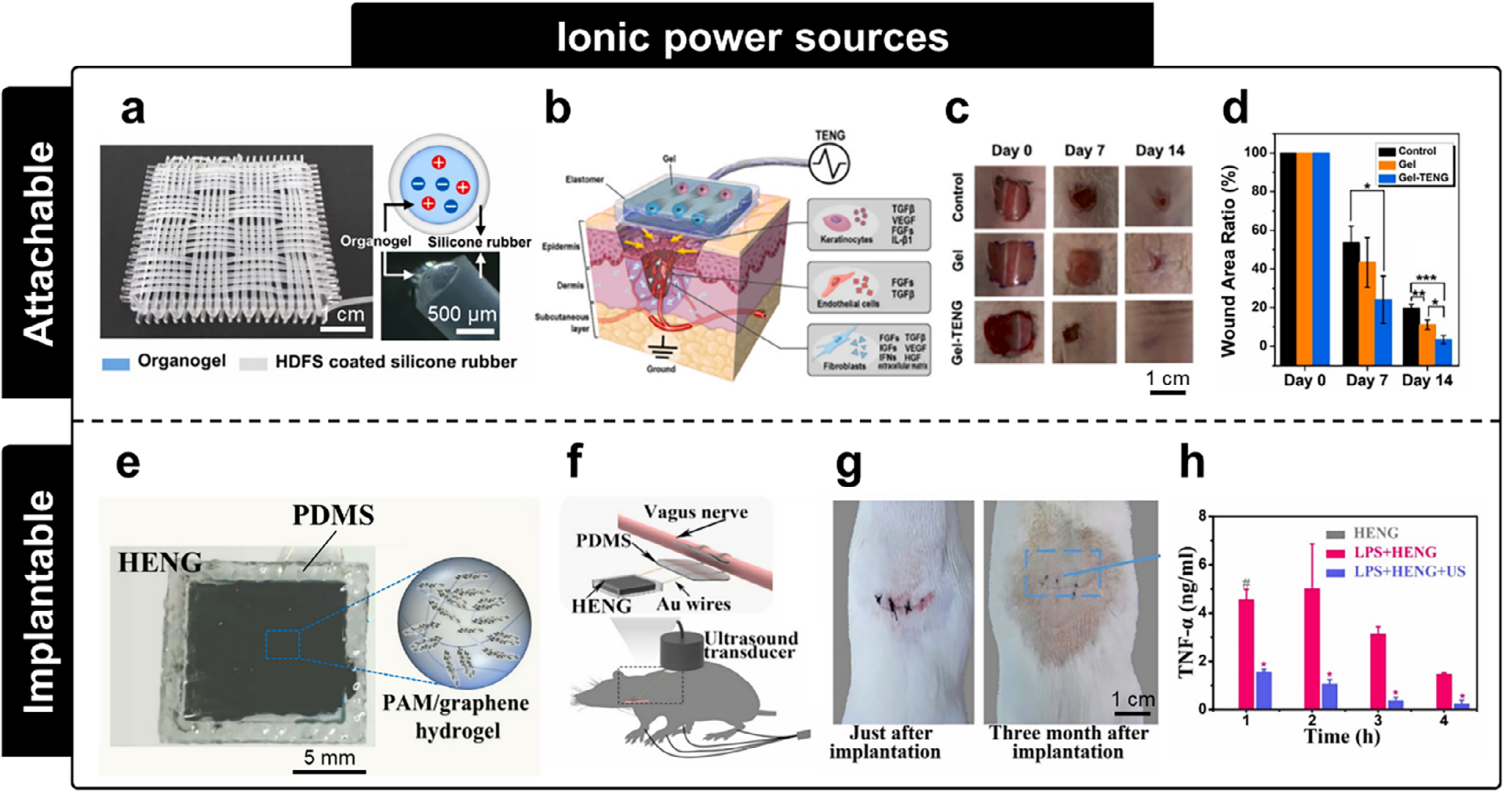
Ionic TENG‐based power sources for attachable/implantable biomedical applications. (a) Optical image of the ionic fabric (inset: schematic diagram of a cross‐section of the ionic fabric and magnified image of a fiber.). Conductive organogel is injected into a heptadecafluoro‐1,1,2,2‐tetrahydrodecyl trichlorosilane (HDFS)‐treated silicone tube. Reproduced with permission [52]. Copyright 2021, Elsevier. (b) Schematic depiction of accelerated wound healing due to the secretion of biological molecules and the formation of new cutaneous tissue under a self‐powered electric field (EF) driven by an ionic triboelectric nanogenerator (iTENG) patch. (c) Macroscopic appearance of incised wounds in the untreated (control), ionic patch only (Gel), and ionic patch connected with TENG (Gel‐TENG) groups at different times. (d) Graph of the wound area remaining after 3, 7, 10, and 14 days of treatment (*n*  =  3) (*p < 0.05, **p < 0.01, ***p < 0.001). (e) Schematic structure of the high‐performance hydrogel nanogenerators (HENG). Reproduced with permission [128]. Copyright 2021, Elsevier. (f) Schematic illustration showing the process of externally applying ultrasound to a subcutaneously implanted HENG and flexible electrodes wrapped around the vagus nerves, with the aim of suppressing pro‐inflammatory cytokines. (g) Photographs showing the initial state of subcutaneous implantation site after suture and the recovery three months post implantation. (h) Graph showing the concentration of tumor necrosis factor‐α (TNF‐α) in each group over time.

For externally attached biomedical systems, ionic TENGs serve as power supply units that efficiently convert biomechanical energy from surface movements, such as joint motion, muscle contraction, external touch, and friction caused by clothing [121]. As shown in Figure 6a–d, this section introduces accelerated wound healing with an ionic patch assisted by a TENG [52]. This attachable ionic patch, composed of gel‐based and fully stretchable components, functions as both a wound dressing and an electrode, and harvests biophysical energy to generate a uniform electric field that promotes cell migration, proliferation, and growth factor secretion, thereby accelerating wound healing. Figure 6a shows the structure of the ionic triboelectric nanogenerator (iTENG) patch. The iTENG patch consists of a textile‐type iTENG and an ionic wire. Both components are constructed using a polyacrylamide‐based organogel infused with lithium chloride (LiCl), where acrylamide (AAm) forms the polymer network and LiCl provides ionic conductivity [122]. The organogel is injected into silicone microtubes treated with heptadecafluoro‐1,1,2,2‐tetrahydrodecyl trichlorosilane (HDFS) to enhance contact electrification. These ionically conductive and stretchable fibers are woven into a fabric structure to form the textile‐type iTENG, while their unwoven form serves as ionic wires. Figure 6b shows the working mechanism of the iTENG patch. The textile‐type iTENG generates periodic electrical energy through frictional interaction with skin tissue [123]. This energy is transmitted via the ionic wire and induces transient but cyclical charge separation, thereby maintaining an electrical potential difference between the patch and the wound site. At the cellular level, the movement of charged ions through ion channels in the epidermis is induced by electrical stimulation, and the disruption of the transepithelial potential generates an endogenous electric field (EF) that guides dermal cells such as keratinocytes, endothelial cells, and fibroblasts to migrate from the wound edge toward the center [124, 125, 126]. At the molecular level, iTENG‐driven electrical stimulation is also involved in enhancing the secretion of biological molecules such as transforming growth factor (TGF), vascular endothelial growth factor (VEGF), fibroblast growth factor (FGF), and epidermal growth factor (EGF) [127]. This occurs through the activation of various intracellular signaling pathways, including phosphorylation of extracellular signal‐regulated kinases, which are generally known to promote electrostatic cell migration and proliferation. Figure 6c shows the experimental validation of the patch. It illustrates the actual application of the ionic patch (Gel) connected with TENG (Gel‐TENG) group to the wounds of rats. The experiment was conducted using three groups: the control group, the Gel group, and the Gel‐TENG group, and the healing progress was monitored over a period of 14 days. Figure 6d shows the performance of the patch. When the Gel‐TENG patch was applied to the wounds of rats, the wound area ratio defined as the current wound area relative to the initial wound area was approximately 53% in the control group, 43% in the Gel group, and 24% in the Gel‐TENG group on day 7. On day 14, the wound area ratio was approximately 20% in the control group and 10% in the Gel group, while the Gel‐TENG group showed a significantly reduced wound area of around 5%. These results indicate that the application of the Gel‐TENG patch significantly accelerated the wound healing process.

Ionic TENG‐based power sources for implantable biomedical applications harvest energy from various types of biomechanical motions when implanted inside the body, including heartbeats, vascular pulsation, diaphragmatic motion during breathing, and gastrointestinal peristalsis, as well as from externally applied stimuli such as ultrasound or magnetic fields [57]. As shown in Figure 6e–h, this section introduces wireless electrical stimulation of the vagus nerves by ultrasound‐responsive programmable hydrogel nanogenerators for anti‐inflammatory therapy in sepsis [128]. The stimulation was achieved by externally applying ultrasound to the subcutaneously implanted high‐performance hydrogel nanogenerators (HENG) connected to flexible electrodes, leading to the suppression of pro‐inflammatory cytokines and alleviation of systemic inflammation. Figure 6e shows the structure of the HENG. The HENG consists of a PDMS encapsulation framework and a polyacrylamide (PAM)/graphene conductive hydrogel. The PAM/graphene hydrogel is prepared by incorporating graphene into the PAM matrix, where acrylamide forms the polymer network and graphene provides electrical conductivity. A 3D‐printed sacrificial polylactide (PLA) mold is used to shape the PDMS encapsulation framework, which is subsequently cured and treated with methylene chloride to remove the PLA. The hydrogel precursor is then cast into the PDMS framework, and a gold wire coil (100 µm diameter) is embedded as a collection electrode. The device is further encapsulated with a thin PDMS film. Figure 6f shows the working mechanism of the HENG. The device is subcutaneously implanted and connected to flexible electrodes that are wrapped around the cervical vagus nerves. Upon exposure to externally applied ultrasound, the HENG harvests acoustic energy through the vibration‐induced charge displacement at the hydrogel–electrolyte interface, generating alternating electrical pulses. These pulses are delivered to the vagus nerves via the electrodes, resulting in neuromodulation that activates anti‐inflammatory pathways. This wireless electrical stimulation leads to a significant suppression of pro‐inflammatory cytokines and alleviation of systemic inflammation. Figure 6g shows the experimental validation of the HENG. To evaluate the long‐term durability in vivo performance, inflammatory response, and suppression effect of the device, the HENG was subcutaneously implanted in rats. Optical images taken immediately after surgery and three months post‐implantation confirmed complete wound healing and stable tissue integration at the implantation site. The electrical output was also found to be maintained under the same ultrasound conditions, demonstrating the long‐term usability of the HENG in biological environments. Figure 6h shows the anti‐inflammatory performance of the HENG. To evaluate its therapeutic effect, the concentrations of pro‐inflammatory cytokines were measured across different experimental groups. Compared to the lipopolysaccharide (LPS) + HENG group, the LPS + HENG + US group showed a significant reduction in the level of tumor necrosis factor‐α (TNF‐α), a key pro‐inflammatory cytokine. Additional experiments further confirmed that other cytokines, such as interferon‐γ (IFN‐γ) and interleukin‐1β (IL‐1β), were also significantly reduced. These results indicate that ultrasound‐activated HENG effectively stimulates the vagus nerves and suppresses systemic inflammation.

Together, these examples highlight the unique capability of ionic TENG‐based power sources to seamlessly integrate with soft tissues while enabling autonomous, wire‐free electrical stimulation, thereby expanding their applicability for chronic therapeutic modulation in vivo [52].

### 3.2 Ionic TENG‐Based Sensors for Attachable/Implantable Biomedical Applications

**FIGURE 7:**
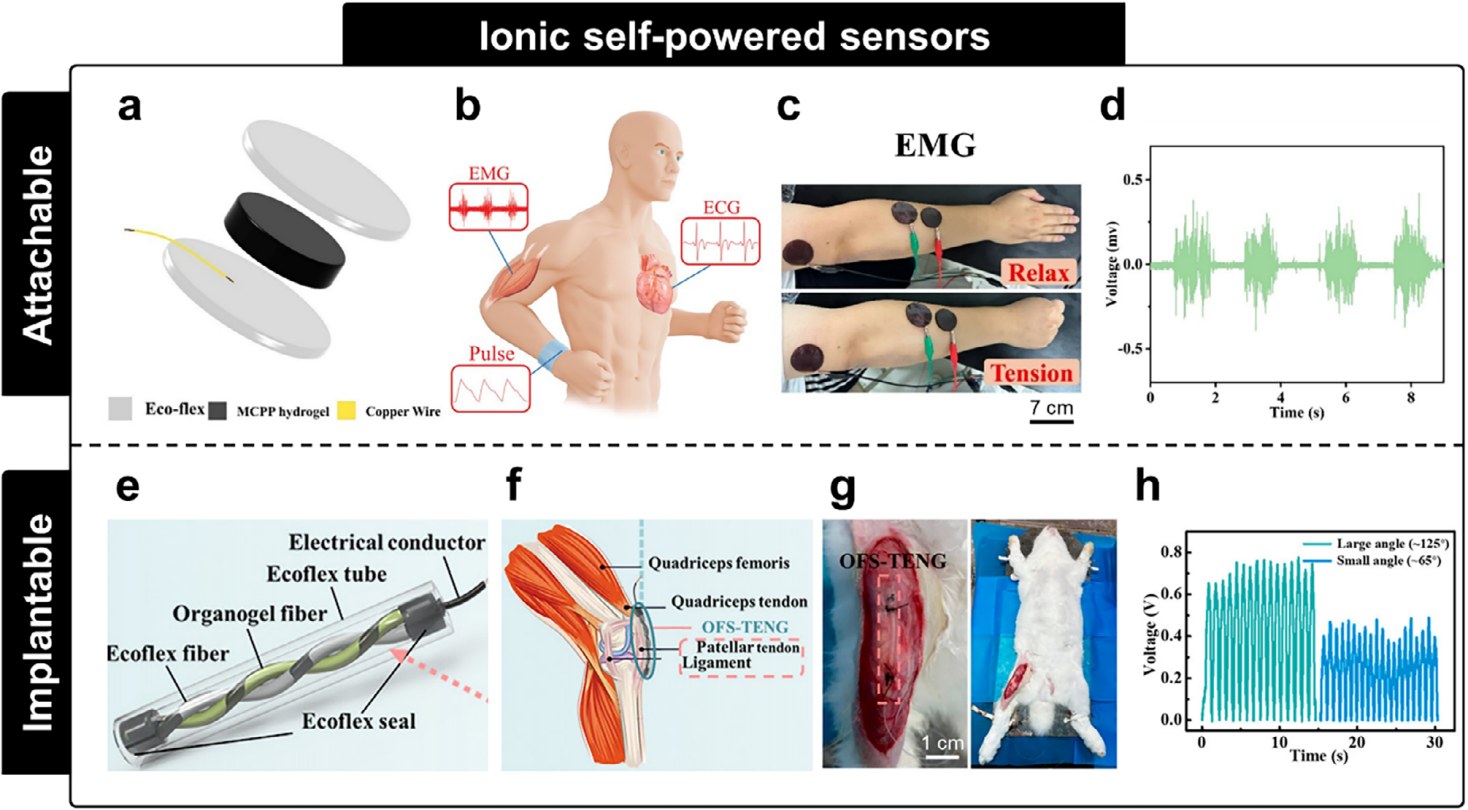
Ionic TENG‐based self‐powered sensors for attachable/implantable biomedical applications. (a) Schematic diagram of the MXene/CNF/PDA/PAM (MCPP) generator. Reproduced with permission [131]. Copyright 2025, Elsevier. (b) Illustration of the working mechanism of the MCPP generator for self‐powered sensing, with electromyography (EMG) and electrocardiography (ECG) signals measured by attaching the device to the forearm, wrist, and ankle. Reproduced with permission [133]. Copyright 2022, Wiley‐VCH. (c) MCPP hydrogel sensor attached to the forearm for EMG signal measurement during fist clenching. (d) EMG signals recorded during repeated cycles of fist clenching and releasing. (e) Architecture of the organogel/silicone fiber‐helical sensor based on a triboelectric nanogenerator (OFS‐TENG). Reproduced with permission [58]. Copyright 2022, The American Chemical Society. (f) Magnified schematic of the sensor implantable in the knee. (g) Photographs of the OFS‐TENG implanted on the patellar ligament of the rabbit knee. (h) Comparison of the output of the implanted OFS‐TENG under repeated bending and stretching cycles of the rabbit leg at different bending angles.

Ionic TENG‐based sensors for attachable biomedical applications are self‐powered sensors that, when attached to the skin, harvest energy from various types of subtle physiological signals and biomechanical movements such as pulse, respiration, voice‐induced vibration, muscle motion, and joint motion, and convert the resulting generated voltage into quantitative data [53, 129, 130]. As shown in Figure 7a–d, this section introduces highly stretchable, self‐adhesive, conductive, and tough hydrogel reinforced with cellulose nanofibers for wearable biosensor and ultrasonic nanogenerator [131]. This hydrogel sensor can be used for detecting human physiological signals (e.g., electrocardiography and electromyography) as well as handwriting recognition, and it offers a novel approach to energy harvesting by generating stable voltage under ultrasonic stimulation. Figure 7a shows the structure of the MXene/cellulose nanofibers (CNFs)/polydopamine (PDA)/PAM (MCPP). The MCPP hydrogel comprises a physically and chemically crosslinked network of polyacrylamide, cellulose nanofibers, polydopamine, and MXene nanosheets. The CNFs enhance mechanical strength via hydrogen bonding with the PAM matrix, while MXene nanosheets establish a continuous conductive path and participate in interfacial interactions with PDA and PAM [56]. PDA formed from dopamine oxidation, introduces mussel‐inspired catechol groups that impart strong self‐adhesion. The resulting 3D porous architecture enables efficient ion/electron transport and mechanical robustness, which is further protected by Ecoflex encapsulation and triboelectrically enhanced with a PA6 film. Figure 7b shows the working mechanism of the MCPP hydrogel. The MCPP hydrogel possesses an efficient internal conductive network formed by MXene nanosheets, which provides excellent electrical conductivity. Owing to this high conductivity, it can detect human bioelectrical signals such as electrocardiography (ECG) and electromyography (EMG) when attached to the skin, generating voltage signals that are utilized for physiological signal monitoring [132]. The resulting voltage waveforms enable the identification of ECG components (P, Q, R, S, and T waves) and the analysis of EMG patterns associated with muscle contractions, allowing real‐time detection of body movements. This allows for noninvasive and continuous physiological signals monitoring without the need for conductive gels or metal electrodes. In addition, the MCPP exhibits self‐powering functionality under external ultrasonic stimulation. When exposed to ultrasound, water within the hydrogel vibrates and flows through MXene‐based microchannels, disrupting the EDL and generating streaming vibration potential, which induces alternating current. Simultaneously, high‐frequency friction between the MCPP surface and the attached PA6 film leads to a triboelectric effect, further enhancing the voltage output. Figure 7c shows the experimental validation of the MCPP hydrogel. The MCPP hydrogel was attached to the forearm to measure EMG signals in practice. In addition, experiments were also conducted by attaching it to the wrist and ankle to detect ECG signals, and to the fingers to detect bending motions. Figure 7d shows the sensing performance of the MCPP hydrogel. It was confirmed that the bioelectrical signals generated during repeated clenching and releasing of the fist were detected through the MCPP hydrogel attached to the forearm, resulting in voltage differences. Repetitive hand movements produced similar voltage waveforms, demonstrating that electromyography signals can be measured stably.

Ionic TENG‐based sensors for implantable biomedical applications harvest energy from various types of biomechanical motions when implanted inside the body, including heartbeats, vascular pulsation, diaphragmatic motion during breathing, and gastrointestinal peristalsis, and convert the resulting generated voltage into quantitative data. As shown in Figure 7e–h, this section introduces ultrastretchable organogel/silicone fiber‐helical sensors for self‐powered implantable ligament strain monitoring [58]. The organogel/silicone fiber‐helical sensor based on a triboelectric nanogenerator (OFS‐TENG) is composed of double‐helical organogel and silicone fibers, offering excellent stretchability (up to 600%), long‐term stability, and biocompatibility, enabling real‐time, power‐free monitoring of ligament strain in vivo. Figure 7e shows the structure of the OFS‐TENG. The device is fabricated by first preparing a conductive organogel fiber via UV curing of a precursor solution dissolved in propylene carbonate, which is composed of 4‐acryloylmorpholine, lithium bis(trifluoromethylsulfonyl)imide, and poly(ethylene glycol) diacrylate, with 1‐hydroxycyclohexyl phenyl ketone added as a photoinitiator. The resulting organogel fiber exhibits excellent stretchability and electrical conductivity. This fiber is helically twisted together with a silicone fiber, and the double helix is inserted into a silicone tube for encapsulation. A copper wire is fixed to the organogel fiber to enable electrical signal output. Figure 7f shows the working mechanism of the OFS‐TENG, which operates beneath the patella and is aligned along the patellar ligament. The working mechanism of the OFS‐TENG is based on the vertical contact‐separation mode of a triboelectric nanogenerator. During mechanical deformation such as stretching or bending, the organogel and silicone fibers within the double‐helical structure are brought into repeated contact and separation. As the ligament or muscle contracts and extends, this deformation compresses and stretches the encapsulated helix, increasing the contact area between the two fibers. Upon contact, triboelectric charges of opposite polarity are generated at the interface. When separated, a potential difference is formed, driving electrons through the external circuit via the attached copper wire. The resulting alternating current correlates with the degree and frequency of mechanical strain. Implanted onto the patellar ligament, the OFS‐TENG sensitively monitors ligament elongation and muscle movement during joint flexion and extension, enabling real‐time strain sensing in vivo. Figure 7g shows the experimental validation of the OFS‐TENG. The sensor was implanted onto the patellar ligament of a rabbit knee to monitor ligament deformation during joint movement. The images show the placement of the sensor and its conformity with the surrounding soft tissue. Figure 7h shows the performance of the OFS‐TENG. To evaluate its sensing performance, the rabbit leg was repeatedly bent at a constant speed to two angles, approximately 125 degrees and 65 degrees. The resulting voltage signals showed a clear dependence on the bending angle, with smaller angles (that is, deeper flexion) producing significantly higher outputs. This increase in output is due to the greater compressive deformation of the helical structure during deep flexion, which increases the contact area between the organogel and the silicone fibers, thereby amplifying the triboelectric response. This indicates that the OFS‐TENG is capable of measuring changes in knee ligament strain caused by movement.

These representative examples demonstrate that ionic TENG‐based sensors can function as self‐powered, highly compliant platforms for real‐time monitoring of physiological signals and tissue mechanics in both external and internal biomedical environments.

### 3.3 Current Challenges in Ionic TENGs for Attachable/Implantable Biomedical Applications

Despite notable progress in this field, the development of biocompatible and stable adhesion mechanisms remains a critical challenge for ensuring long‐term operational stability under dynamic physiological conditions. In attachable systems, conventional chemical adhesives, such as medical tapes, are commonly used for device fixation. However, these chemical agents often trigger allergic reactions or contact dermatitis, and their performance significantly degrades due to sweat accumulation during prolonged wear. In implantable systems, fixation typically relies on surgical suturing or subcutaneous insertion. While functional, suturing can cause localized tissue damage and chronic inflammation, whereas subcutaneous placement is prone to adhesion failure in the body's moist and dynamic environment. These issues make long‐term in vivo operation particularly challenging, as chronic inflammation and the gradual loss of interfacial adhesion often limit existing studies to short‐term validation (typically several weeks or months).

Overcoming these interfacial and fixation issues necessitates biocompatible and stable anchoring methods. As a potential solution, we propose mechanical adhesion strategies utilizing biocompatible materials. For instance, bio‐inspired mechanisms, such as octopus‐inspired suction cups, offer reliable and reversible adhesion without compromising tissue integrity. Furthermore, unlike chemical adhesives or surgical suturing, this approach minimizes adverse tissue reactions and inflammatory burdens, highlighting its potential to facilitate stable, long‐term implantation.

### 3.4 Summary of Ionic TENGs for Attachable/Implantable Biomedical Applications

This section summarizes ionic TENG‐based systems designed for attachable and implantable biomedical applications, serving as both sustainable power sources and self‐powered sensors for physiological monitoring. Representative demonstrations include skin‐conformable patches for motion sensing and internally fixed devices for monitoring organ dynamics. The previous section addressed the inherent limitations of conventional chemical and invasive fixation methods and proposed bioinspired physical adhesion as a solution. Through these considerations, the technical transition from irritant‐prone chemical adhesives to biocompatible, bioinspired physical adhesion mechanisms will serve as a key strategy to bridge the gap between robust interfacial stability and long‐term biological safety.

## 4 Conclusions and Future Directions

We have summarized ionic‐ and electronic TENG‐based sustainable power sources and self‐powered sensors for biomedical applications, emphasizing their underlying materials, device architectures, and working mechanisms. As discussed in the preceding sections, advancements in soft ionic and electronic materials have enabled rapid progress in replacing conventional portable power sources with TENGs, offering promising solutions to the limitations of operational lifetimes and frequent battery recharging.

However, a closer examination of attachable and implantable systems throughout this review reveals several persistent limitations that must be addressed to facilitate their general utilization in daily life. Specifically, the instability of skin–device adhesion under sweat exposure is identified as a major bottleneck in long‐term attachable applications (Section 3.3), necessitating the development of sweat‐resistant interfaces. In addition, despite the structural design strategies reviewed for stretchability, mechanical mismatch and material fatigue remain unresolved due to the intrinsic rigidity of electronic components (Sections 2.3 and 3.1), underscoring the importance of highly skin‐compliant materials. In this context, the incorporation of AI‐enabled design and control strategies becomes essential to rationally optimize material composition, interfacial adhesion, and device architecture across dynamic and highly variable physiological conditions. Furthermore, while ionic materials enable acoustic transparency and imaging compatibility in implantable devices (Section 3), their inherent invisibility presents challenges in device localization, thus necessitating new strategies for imaging‐aware integration. Finally, although implantable TENG systems demonstrate effective in vivo functionality, their reliance on surgical implantation and fixation remains a critical barrier (Sections 3.1, 3.2 and 3.3), emphasizing the need for minimally invasive and cut‐free implantation approaches. Beyond technical performance, a systematic analysis of clinical translation barriers, including regulatory compliance, sterilization compatibility, and scalable fabrication of human‐sized devices, is indispensable for aligning emerging TENG technologies with the practical requirements of clinical implementation.

**FIGURE 8:**
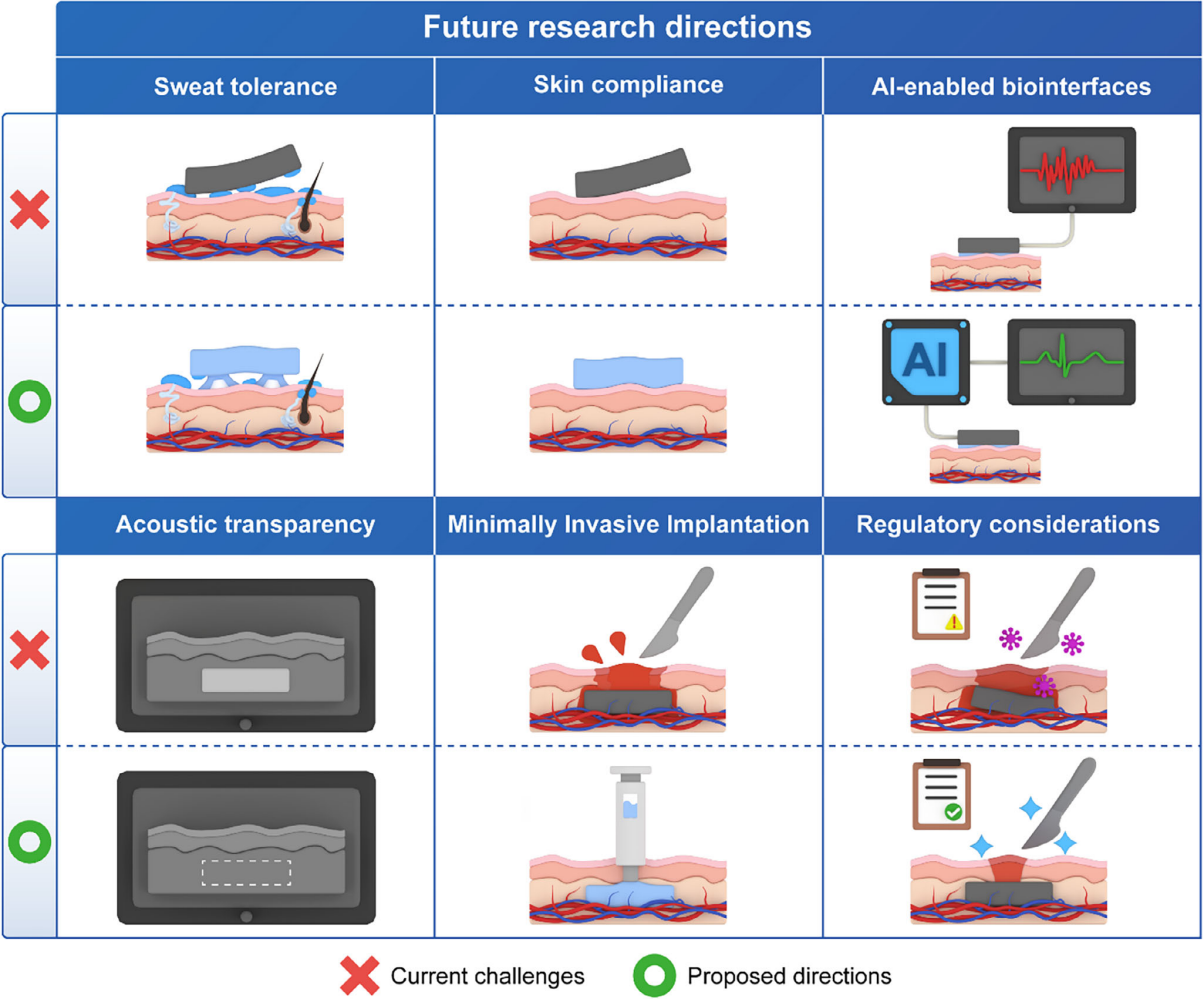
Future research directions for biomedical devices. Future biomedical TENG applications are expected to integrate six key aspects: sweat tolerance strategies, skin‐compliant deformation of intrinsically soft materials, AI‐assisted signal processing, acoustic transparency of implantable devices, minimally invasive implantation via injection, and regulatory considerations for clinical translation.

Accordingly, this section outlines future research directions that directly respond to these identified challenges, focusing on six key aspects: sweat tolerance, skin compliance, AI‐enabled TENG biointerfaces, acoustic transparency, minimally invasive implantation strategies, and regulatory considerations for clinical translation (Figure 8).

### 4.1 Sweat Tolerance

Sweat tolerance of attachable devices at the skin interface is essential for long‐term applications. However, sweat‐induced deterioration of adhesion remains a critical challenge to ensuring device stability. For instance, chemical adhesives are commonly applied to attach devices to the skin. However, exposure to sweat or body fluid can promote adhesive degradation and residue transfer, leading to surface contamination and side effects such as allergies and rashes. Moreover, they can suffer a degradation of adhesion strength when reattached to the skin. To overcome these limitations, mechanical adhesion strategies inspired by nature have gained significant attention [134]. For example, the octopus provides inspiration for adhesion strategies that offer minimal adverse reactions and high reusability. The integration of biomimetic strategies into biomedical devices could achieve stable, non‐chemical adhesion on skin.

To further address interfacial failure, researchers have pursued two distinct structural engineering approaches. One approach focuses on sweat‐permeable interface designs that allow natural perspiration while maintaining stable device attachment [135, 136]. For example, a sweat pore–inspired perforated electronic skin (e‐skin) was developed to prevent sweat accumulation at the skin interface, thereby mitigating sensor malfunction and delamination. By incorporating auxetic dumbbell‐shaped through‐hole patterns, the e‐skin enables efficient sweat permeation while simultaneously enhancing mechanical flexibility, conformability, and adhesion. Such breathable architectures maintain stable sensing performance even under intense sweating or prolonged physical activity, highlighting an emerging strategy for preserving skin homeostasis in long‐term wearable and attachable devices.

The other approach involves mechanically driven, bioinspired adhesion strategies, such as the octopus‐inspired adhesion mentioned previously, which continue to be widely investigated as a robust alternative to chemical adhesives for achieving strong, reusable adhesion on wet and deformable surfaces without chemical bonding. More recently, remora fish–inspired systems have further extended this concept toward extreme physiological environments [137]. Inspired by the adhesive disc of the remora fish, the Mechanical Underwater Soft Adhesion System (MUSAS) was developed to achieve robust attachment to dynamic, wet, and compliant substrates, such as gastrointestinal tissues. By combining mechanically adaptive lamellar structures with soft elastomeric suction interfaces, MUSAS demonstrates stable adhesion across substrates with diverse stiffnesses and surface roughness, without relying on chemical adhesion or prolonged pre‐attachment pressure.

Collectively, these advances illustrate a broader trend toward structurally engineered, bioinspired adhesion platforms that effectively address sweat, moisture, and tissue dynamics at the interface. Such strategies enable transformative biomedical applications, including long‐term physiological monitoring, non‐invasive gastrointestinal sensing, and localized drug delivery. Overall, bioinspired mechanical and sweat‐permeable adhesion designs represent a promising future direction for achieving stable, long‐term attachment of biomedical devices under challenging physiological conditions.

### 4.2 Skin Compliance

Mechanical compliance is a critical property for skin‐mounted devices, as human skin exhibits a low Young's modulus and is capable of large deformations. However, conventional devices are generally fabricated with rigid materials that show intrinsically limited deformability, thus leading to a mechanical mismatch with the dynamic deformation of skin. To overcome this limitation, researchers have explored various structural designs (e.g., wrinkles, serpentine patterns, and origami mechanics) to incorporate stretchability into devices [30]. Despite significant progress in electronics, material fatigue induced by plastic deformation and generally low range of strain of the brittle electronic materials remain the inevitable challenges. Replacing such rigid materials with intrinsically soft and deformable ionic materials could enable the creation of devices compliant with skin. For instance, soft material‐based devices, such as ionic skins fabricated using hydrogel [138], demonstrate a touch‐sensing capability even when highly deformed while attached to the skin. In the future, these soft ionic devices will offer new ways to minimize the mechanical mismatch with skin, thereby enabling the development of skin‐conformal interactive devices.

### 4.3 AI‐Enabled Biointerfaces

Research on soft ionic and electronic TENGs continues to yield various advantageous findings, with researchers particularly striving to further enhance their portability, wearability, attachability, and implantability. However, the inherent limitations of TENG materials and the challenges associated with their large‐scale clinical integration still remain clear [139]. For these devices to be routinely adopted in clinical environments, optimization must likely address complex, multivariate performance indicators, considering factors like the size of the affected area and the unique physiology of different organs [140].

In TENG‐based biointerfaces, device performance emerges from tightly coupled nonlinear interactions among mechanical deformation, surface charge dynamics, ionic mobility, and biological variability. Such complexity makes conventional trial‐and‐error optimization of materials and structures impractical [141]. Rather than attempting to overcome these intrinsic limitations directly, AI reframes them as learnable system parameters, seeking to achieve robust performance and functional reliability even under non‐ideal biological conditions. By leveraging AI to learn from experimental, simulation, and physiological data in a feedback loop, TENG‐based biointerfaces can achieve intelligent, self‐adaptive, and anticipatory functionality.

The recent, rapid advancement of AI technology, specifically Machine Learning and Deep Learning, offers a potential opportunity to navigate this multidimensional parameter space. AI‐driven frameworks can be leveraged to perform advanced variable control, predicting optimized conditions by learning from both experimental and physiological data. By applying virtually derived results to real‐world applications and reflecting them back into the AI, researchers can secure significant datasets through a back‐to‐back data construction process. Beyond material and structural optimization, AI may hold significant strength, particularly in processing the complex electrical outputs of TENGs [142, 143, 144]. AI enables the translation of raw triboelectric signals into clinically meaningful physiological states, thereby facilitating a transition from passive sensing toward predictive biointerfaces. This functionality might also extend beyond simple signal denoising to enable adaptive closed‐loop therapy.

Overall, the convergence of TENG technology with AI introduces a new research axis that fundamentally extends beyond material and structural optimization. AI‐enabled TENG‐based biointerfaces represent a compelling step toward intelligent, self‐adaptive, and forecasting biomedical systems capable of personalized diagnostics and closed‐loop therapy, which may pave the way for next‐generation human–machine interfaces.

### 4.4 Acoustic Transparency

The unique properties of ionic materials, particularly their optical and acoustic transparency, offer innovative advantages for next‐generation implantable applications. Hydrogels, for instance, are composed of hydrophilic polymer networks and water (60–90 wt%), which results in high optical transparency due to minimal light scattering from the polymer network [145]. Especially, in an aqueous environment, they exhibit acoustic transparency due to an acoustic impedance match [146]. This invisibility makes hydrogel‐based devices ideal for passive camouflage applications [147, 148]. While this has been explored in attachable and underwater applications, their acoustic transparency has significant potential for the development of invisible implantable devices [148]. When these materials are applied to implantable devices, this camouflage property minimizes the influence of artifacts in diagnostic imaging, with the potential to enhance image clarity. However, this invisibility could present a challenge for monitoring the implanted position of the device within the body. In the future, a key research direction would be the integration of minimal markers into implantable devices. This approach would enable visualization of the device–tissue interface while minimizing imaging artifacts.

### 4.5 Minimally Invasive Implantation

Recent medical efforts have focused on developing minimally invasive surgical procedures to minimize wounds, shorten surgical time, and reduce anxiety in patients [149]. To achieve this, several design paradigms, including miniaturization, injectable systems, and biodegradability, are essential for the next generation of biomedical devices.

Miniaturization is a critical design requirement for next‐generation devices [150]. Reducing the size and thickness of the device minimizes insertion‐induced tissue damage and inflammatory responses, thereby substantially shortening patient recovery time. By enabling device dimensions to native tissue microstructures, the mechanical mismatch with surrounding tissue is reduced, leading to decreased inflammation and fibrosis and promoting the formation of a stable, long‐term tissue–device interface. Furthermore, ultrathin and ultrasmall devices exhibit enhanced mechanical compliance, allowing them to deform synergistically with soft tissues, which minimizes foreign‐body sensation and mechanical interference during daily activities [151].

From a systems perspective, device miniaturization is key to achieving efficiency. Reduced circuit and sensor dimensions inherently lower power consumption, which is critical for enabling long‐term operation using compact energy storage or self‐powered TENGs. Moreover, miniaturized architectures enable the seamless integration of multichannel sensing, wireless communication, and feedback control within a small footprint. Complementary to miniaturization, injectable devices represent a promising strategy for minimally invasive surgery, as they minimize the need for incisions [152]. For example, an injectable solution could be cured in vivo by various stimuli such as light, temperature, or pH, enabling its biomedical applications such as wound healing and drug delivery [153, 154]. Moreover, utilizing in situ gelation techniques to integrate a dielectric layer and an ionic conductor could pave the way for self‐powered sources or sensors based on an ionic TENG mechanism [155]. This ‘cut‐free’ implantation method based on injection offers the benefits of minimizing scarring, reducing infection risk, and accelerating patient recovery.

Ultimately, coupling extreme miniaturization with controllable biodegradability represents the long‐term goal for advanced biomedical devices [156]. Enabling implanted systems to naturally resorb after completing their intended function eliminates the need for secondary surgical removal, further reducing patient burden and significantly improving long‐term biocompatibility. Together, these miniaturized, injectable, and biodegradable device platforms offer a promising pathway toward minimally invasive, patient‐compliant, and clinically translatable biomedical technologies.

### 4.6 Translational Barriers and Regulatory Considerations for TENGs

Clinical translation of TENG technologies still faces a number of unresolved practical and regulatory issues. One of the most pressing challenges is scaling. To date, nearly all experiments have been performed in small‐animal models, leaving the behavior of larger, human‐scale systems uncertain. When scaled up, the materials must withstand significantly greater mechanical stress and repeated deformation without compromising output performance or long‐term stability. To address these durability concerns, two essential standardized testing frameworks are utilized. First, tests adapted from the IEC 60068‐2 series (environmental stress, vibration, temperature cycling) [157] enable the standardized evaluation of environmental stability in TENG materials under physiologically relevant temperature and humidity conditions. In parallel, tests inspired by ASTM F2077 (mechanical fatigue of biomedical implants) [158] provide a clinically grounded framework to assess cyclic mechanical fatigue and long‐term durability under repetitive physiological loading. By simulating these cyclic conditions in a controlled setting, researchers can accurately predict the lifetime performance and clinical viability of human‐scale TENG devices.

A second major concern is sterilization. Many soft ionic or electronic‐based TENGs are reported to be vulnerable to mechanical deformation or loss of charge‐transfer ability during standard high‐temperature steam sterilization. Consequently, alternative low‐temperature methods, such as ethylene‐oxide (EtO) or gamma irradiation, must be optimized under the guidance of ISO 11135 and ISO 11737 to protect device functionality and ensure the absence of residual gases [159, 160, 161]. This necessity for alternative methods has led to comparative studies [162]. For example, one study compared multiple sterilization methods for gelatin methacryloyl (GelMA) hydrogels, including UV irradiation, ethanol treatment, and autoclaving, to specifically evaluate their effects on the material properties and long‐term stability. They proved that these irradiation‐based sterilization methods could pose a risk. They may induce subtle structural changes at the material level or alterations in charge transfer properties, thereby potentially impacting the functional stability of TENGs. While these effects may not be clearly manifested as short‐term output changes, they can act as critical variables in terms of long‐term reliability under repetitive operation conditions.

For regulatory clearance, TENG devices will ultimately need to align with both FDA (Food and Drug Administration) and CE (Conformité Européenne) expectations. Implantable or self‐powered stimulators in Europe must comply with the Medical Device Regulation (MDR, EU 2017/745), with harmonized standards such as EN 45502 providing technical guidance for active implantable medical devices [163]. Simultaneously, ISO 10993 biological evaluations are critical, including key parts such as Part 5 (in vitro cytotoxicity), Part 10 (irritation and sensitization), Part 17 (allowable limits for leachable substances), and Part 18 (chemical characterization of materials) [164, 165, 166, 167, 168]. Electrical and electromagnetic safety must comply with IEC 60601‐1 and IEC 60601‐1‐2 [169, 170]. Furthermore, the ASTM F3144 guideline [171] can be used to rigorously examine energy‐storage stability and residual electrical risk during long‐term operation. Aligning these data at an early stage within a structured risk management framework [172] following ISO 14971 and a quality management system compliant [173] with ISO 13485 helps guide regulatory planning and ensures that potential safety issues are addressed proactively. As TENG‐based implantable platforms continue to evolve, examining their regulatory relevance to established AIMD frameworks (e.g., ISO 14708‐3) may help inform future design, testing, and clinical translation strategies.

Another barrier is stable tissue fixation. Most reported TENGs rely on sutures to stay in place, which can cause micro‐motion and poor electrical contact in vivo. To ensure stable adhesion to biological tissues, bio‐inspired design strategies could be employed. For instance, incorporating features like the suction cup structures of octopi, micro‐patterned adhesive structures of geckos, or microneedle‐like surfaces of roses can enhance adhesion strength. The adhesion performance of these TENG devices can be quantitatively tested using peel‐off measurements following ASTM D903 [174], while stability at the tissue fixation interface under mechanical stimuli can be evaluated according to ASTM D1876 (delamination test) [175].

In summary, the successful clinical translation of implantable TENGs hinges on the integration of four critical elements: robust mechanical reliability (IEC 60068, ASTM F2077), validated safe sterilization (ISO 11135, ISO 11737), verified biocompatibility and electrical safety (ISO 10993, IEC 60601), and comprehensive long‐term stability assessment (ASTM F3144). With this regulatory and technical foundation, TENGs can evolve from experimental energy harvesters into clinically dependable self‐powered bioelectronic systems capable of supporting next‐generation therapeutic implants.

## Conflicts of Interest

The authors declare no conflicts of interest.

## Data Availability

The authors have nothing to report.
